# Bleomycin-Electrosclerotherapy in the treatment of superficial slow-flow vascular malformations of head and neck

**DOI:** 10.3389/fneur.2026.1826764

**Published:** 2026-04-13

**Authors:** Florian Obereisenbuchner, Caroline T. Seebauer, Elena Borisch, Daniel Puhr-Westerheide, Sinan Deniz, Alena Sint, Natascha Platz Batista da Silva, Veronika Vielsmeier, Thomas Kühnel, Felix H. Vollbach, Julia Haehl, Alexandra Hartl, Walter A. Wohlgemuth, Jens Ricke, Max Seidensticker, Moritz Wildgruber, Vanessa F. Schmidt

**Affiliations:** 1 Department of Radiology, LMU University Hospital, LMU Munich, München, Germany; 2 Interdisciplinary Center for Vascular Anomalies (IZGA), LMU University Hospital, LMU Munich, München, Germany; 3 Department of Otorhinolaryngology, Head and Neck Surgery, Luzerner Kantonsspital, Lucerne, Switzerland; 4 Department of Otorhinolaryngology, Regensburg University Medical Center, Regensburg, Germany; 5 Department for Radiology and Neuroradiology, RoMed Hospital Rosenheim, Rosenheim, Germany; 6 Department of Plastic and Aesthetic Surgery, LMU University Hospital, LMU Munich, München, Germany; 7 Department of Paediatric Surgery, LMU University Hospital, LMU Munich, München, Germany; 8 Clinic and Policlinic of Radiology, Martin-Luther University Halle-Wittenberg, Halle, Germany

**Keywords:** BEST, Bleomycin-Electrosclerotherapy, lymphatic malformation (LM), slow-flow vascular malformation, venous malformation (VM)

## Abstract

**Introduction:**

Recently, Bleomycin-Electrosclerotherapy (BEST) has emerged as a novel alternative in the treatment of slow-flow vascular malformations. Regarding the commonly affected head and neck area, this procedure has to be safe and controllable due to the close anatomical proximity of critical structures. The aim of this study is to evaluate the safety and clinical outcome of BEST for slow-flow vascular malformations of the head and neck region.

**Methods:**

Twenty-five patients with symptomatic predominantly superficial SFVMs were treated by 41 BEST sessions between 11/21 and 09/24. Treatments of lesions involving the aerodigestive tract were excluded. Patient records and imaging findings were analyzed with respect to clinical and procedural details. Changes in overall symptom severity were classified into five categories (complete-, marked-, mild-, no response, and progress) based on patients’ reports. Pre- and postprocedural lesion size was determined by measuring the maximum diameter in three orthogonal planes on MRI. Complications and side-effects including skin discoloration were assessed.

**Results:**

The most common indications for treatment were swelling in 17/25 patients (68%), followed by pain (7/25, 28%), and functional impairment (7/25, 28%). Patients received 1.6 ± 1.1 BEST sessions with a median bleomycin dose of 5 mg (range 1–15 mg). Complete response at the end of follow-up was observed in 8/25 (32%), strong reduction of symptoms in 14/25 (56%), and mild reduction in 3/25 (12%) patients. No patients showed progression or were non-responders. Follow-up MRI revealed a lesion size reduction in all patients for which a follow-up MRI was available and complete or partial reduced contrast uptake in 8/10 (80%) patients. Prolonged pain and swelling were observed after 4/41 (9.8%) of BEST sessions. There were no major complications following the treatment of superficial SFVMs, especially no nerve injury. Skin discoloration was observed in 9/25 (36%) of patients while all patients already reported partially fading during follow-up. All complications were temporary only and subsided at follow-up.

**Discussion:**

BEST is safe and effective for treating slow-flow vascular malformations of the head and neck region with an acceptable risk- and complication profile.

## 1 Introduction

The International Society for the Study of Vascular Anomalies (ISSVA) distinguishes slow-flow and fast-flow vascular malformations dependent on whether the lesion contains arterial components (1, 2). As opposed to vascular tumors which are characterized by an increased proliferation of endothelial cells and might sometimes regress spontaneously with the patient’s age, vascular malformations are based on angiogenetic and mesenchymal disorders and usually do not regress without treatment (3, 4). Slow-flow vascular malformations (SFVMs) such as venous malformations (VMs) and lymphatic malformations (LMs) are regularly treated by percutaneous injection of different sclerosing agents, the most common ones being liquid ethanol, ethanol gel, polidocanol, sodium tetradecyl sulfate, and bleomycin (5–9). Using these, multiple sessions are often necessary particularly regarding microcystic LMs and extensive VMs, making treatments challenging (10). Recently, Bleomycin-Electrosclerotherapy (BEST) has been established as a novel therapeutic approach in the treatment of SFVMs. BEST combines reversible electroporation, resulting in a transient increase in cellular membrane permeability, with intralesional or intravenous application of bleomycin, leading to a thousandfold increase in local intracellular concentration (11, 12). Initial results in the treatment of SFVMs seem promising (13–18). The head and neck area is the region of the body most commonly affected by SFVMs, especially LMs (19). Treatment in this localization is associated with particular safety concerns due to the high density of vulnerable structures such as nerves surrounded by minor soft tissue coverage (20). Regarding the usage of needle electrodes in combination with reversible electroporation, additional caution is required during BEST in the head and neck region. We aimed to investigate the safety and clinical outcome of BEST for superficial SFVMs in this delicate anatomical region.

## 2 Materials and methods

The present retrospective study including two interdisciplinary vascular anomaly centers at tertiary care university hospitals in Germany was approved by the local ethics committee (University Hospital, LMU Munich, protocol No. 21–0943) and performed in accordance with the Declaration of the World Medical Association (WMA). Informed consent was waived due to the retrospective study design. However, informed consent was obtained from all patients with respect to publish clinical and radiological images. Data collection was conducted using a combination of electronic patient records of treated BEST patients and the Picture Archiving and Communication System (PACS) in the respective centers. The diagnosis of SFVM was based on patient history, physical examination, and imaging using both (duplex-)ultrasound (US) and magnetic resonance imaging (MRI). All BEST therapy sessions of mainly superficial (cutaneous, subcutaneous and muscular) SFVMs affecting the head and neck area from 11/21 until 09/24 were included. Lesions affecting primarily the tongue or airways were excluded due to the expected different risk-profile, including the need for, i.e., protective tracheostomy or prolonged intubation on intensive care due to potential compression of the airways (18).

Decisions for BEST as the therapy of choice were made based on interdisciplinary discussions in the local board for vascular anomalies. The main indications for BEST were symptomatic lesions (pain, swelling, bleeding, recurring infections, repetitive thrombosis, thrombophlebitis, and/or consumptive coagulopathy, aesthetic disfigurement as well as accompanying functional impairment). Selection criteria for BEST have been (1) untreated lesions not considered amenable for conventional treatments with percutaneous sclerotherapy such as microcystic LMs and (2) therapy-refractory or recurrent SFVMs, defined as lesions that persist or progress clinically and/or radiologically following previous percutaneous sclerotherapy or surgery. Exclusion criteria for BEST were pregnant or lactating patients or patients who already reached a lifetime dose of 100 mg of bleomycin (or 1.3 mg/kg for children), in line with the updated current operating procedures (COP) for electrosclerotherapy (21). In women of childbearing age, ß-HCG was tested, and patients were informed to avoid conception during the next 6 months after the procedure. As the most feared complication of bleomycin is pulmonary toxicity, all patients were assessed clinically (included history of past pulmonary disease) for acute or chronic lung injury. In case of positive history or clinical finding, pulmonary function tests and chest *x*-ray were obtained.

### 2.1 Procedural details

The treatment was performed under general anaesthesia (total intravenous anaesthesia using Propofol) and TOF 0 relaxation. FiO_2_ was kept below 30%. In case of VMs and macrocystic LMs, the lesion was directly punctured under US-guidance, followed by contrast injection under fluoroscopy-guidance and subsequent bleomycin injection. In case of microcystic LMs, bleomycin was injected interstitially along the lesion under ultrasound guidance. Bleomycin was routinely used at a dilution of 1 mg/mL saline. The injected bleomycin dose was adjusted according to lesion size (longest diameter) and drainage pattern: up to 0.5 mg for lesions <1 cm, 0.5–1 mg for lesions 1–3 cm, 1–2 mg for lesions 3–5 cm, and >2 mg for lesions >5 cm according to the COP for BEST (21). One minute after bleomycin injection, reversible electroporation was performed. Electrodes (finger electrodes and hexagonal electrodes) were selected based on configuration, localization, and depth of the lesion, while the same procedure was repeated at different locations after percutaneous access always using a fraction of the bleomycin solution if needed. The malformation was covered as systematically as possible during pulse application, with electrodes generally positioned sequentially, row by row, to ensure that no relevant areas were missed. Similarly, overlapping electrode placement was avoided to prevent irreversible electroporation and subsequent tissue necrosis. Nerve structures and arterial vessels were actively avoided under ultrasound guidance to prevent mechanical injury and potential temporary or persistent sequelae. If possible, the whole lesion volume was covered. The maximum bleomycin dose accepted was 0.2 mg per kg bodyweight per treatment session and less than 1 mg/kg bodyweight cumulative, therefore less than in previous reports (11). To apply the reversible electroporation, the electroporation system (Cliniporator™ VITAE, IGEA S.p. A., Carpi, Italy) was used, which provides several independently controlled and isolated outputs, each reaching up to 1,000 V/cm (maximum current: 50 A) and generates electrical impulses with a duration of 100 μs between the electrodes. Periinterventional antibiotic single shot treatment was provided in extended lesions after 20 or more applications of electric pulses in line with the COP for BEST (21).

### 2.2 Outcome evaluation

Patients were routinely scheduled for a standardized follow-up regime with clinical follow-up performed 3–6 months after the last BEST session. Additional BEST sessions were performed in case of insufficient improvement of symptoms and residually perfused lesion(s) on imaging. Follow-up included history taking, clinical examination and US. MRI (contrast-enhanced T1w and T2w fat-sat in two planes and DWI/ADC) was acquired after completion of the planned BEST sessions together with the follow-up visit. The assessed clinical parameters were changes in reported and observed pain intensity and frequency, swelling, functional impairment, bleeding/ulceration, cosmetic disfigurement, frequency of recurrent thrombophlebitis or superinfection. Change in symptom severity was classified into five categories (complete response, marked response, moderate response, no response, and progression) based on the documentation of clinic visits. Likert scale grading was performed independently by a senior interventional radiologist (IR) and an IR with 20 and 8 years of experience in the treatment of vascular malformations, respectively. In cases of discrepant category assignment, the cases were reviewed jointly, and a final category was determined by consensus. All reported or documented peri- or postinterventional complications were categorized according to the CIRSE classification system (22). Respiratory surveillance after BEST is routinely performed according to COP (21), and in case a patient developed respiratory symptoms, post-treatment assessment of pulmonary function is performed. Skin discolorations were recorded, and changes were addressed in every subsequent visit. Outcome measurement for imaging response were changes in lesion size (volume calculated by measuring the maximum diameter in three orthogonal planes) and contrast uptake in MRI before BEST compared to postprocedural MRI (partial fibrosis was rated on a five-point Likert scale: no residual contrast uptake, strong reduction, mild reduction, no change, and progressive contrast uptake). Imaging was reviewed by two experienced radiologists specialized in the diagnostics and treatment of vascular malformations. In case of discordance between the two radiologists, a third radiologist was involved to reach consensus.

### 2.3 Statistical analysis

R-Studio (version 2024.09.0 + 375) and R (version 4.3.2) were used for descriptive statistics and statistical testing. Alpha was set to <0.05. The distribution of patients among the different categories was evaluated descriptively. The Shapiro-test was used to assess the normality of data distribution. Data are presented as mean (±standard deviation) if normally distributed or as median (range/minimum–maximum) if the normality assumption was violated. Given the sample size, Wilcoxon signed-rank test was used to compare the imaging results and Chi-squared test was used to assess the clinical response.

## 3 Results

### 3.1 Patient characteristics

A total of 25 patients, 15 females and 10 males, with primarily superficial SFVMs of the head and/or neck who underwent BEST treatments between 2021 and 2024 in two tertiary care hospitals in Germany were included. The most common types of malformation were VMs in 18/25 (72%) cases, followed by LMs in 4/25 (16%), combined capillary-venous malformations (CVMs) in 2/25 (8%), and combined capillary-lymphatic malformations (CLMs) in 1/25 (4%) case. Most common indications for treatment (multiple selection) were swelling, reported by 17/25 (68%), followed by pain (7/25, 28%), and functional impairment (dysphagia, problems with speaking or dyspnoea) in 7/25 (28%) patients, for details see Table 1. The most involved anatomical areas were the cheek (20/25, 80%), followed by mouth/lips (15/25, 60%), for details see Table 1. Some lesions involved two or more regions. Median age at treatment initiation was 23 years (range 2–78 years) with 10/25 (40%) pediatric cases (age <18 years). Both therapy-naïve (9/25, 36%) and patients with prior invasive treatment (16/25, 64%) were included. For the latter, the median interval to the first BEST session was 7 months (range 1–84). The most common invasive treatments were percutaneous sclerotherapy (6/25, 24%), surgical debulking (3/25, 12%), a combination of both (4/25, 16%), and laser treatment (2/25, 8%) as well as one patient with systemic sirolimus without sufficient symptom improvement.

**Table 1:** Patient characteristics.

Total cohort	*n* = 25	[%]
Age at first treatment	23 (2–78)	
Female	15	60
Types of malformations		
VM	18	72
LM	4	16
CVM	2	8
LVM	1	4
Involved anatomical areas		
Cheek	20	80
Mouth/lips	15	60
Neck	6	24
Skull	2	8
Thorax/Mediastinum	2	8
Treatment rationales		
Swelling	17	68
Pain	7	28
Functional impairment	7	28
Bleeding/ulceration	6	24
Cosmetic disfigurement	5	20
Thrombophlebitis/superinfection	4	16
Others	1	4
Prior therapies		
No	9	36
Sclerotherapy only	6	24
Sclerotherapy and surgery	4	16
Surgery only	3	12
Laser	2	8
Sirolimus	1	4

VM, venous malformation; LM, lymphatic malformation; CVM, capillary-venous malformation; LVM, lympho-venous malformation.

### 3.2 Procedure characteristics

The patients underwent a total of 41 sessions of BEST with a mean of 1.6 ± 1.1 procedures per patient (range 1–5), for details see Table 2. The median interval between repeated BEST treatments was 6 months (range 3–13). In total, 19/41 (46.3%) procedures were performed in children. Median bleomycin dose per procedure was 5 mg (range 1–15 mg) and the median number of cycles of reversible electroporation was 14 (range 2–62). Overall median bleomycin dosage per patient was 7 mg (range 1–32.5 mg). Fluoroscopy and US combined were used for image-guidance in 73.2% (30/41) of procedures. In the remaining 26.8% (11/41) procedures, only US-guidance was used.

**Table 2:** Procedural characteristics.

Per therapy session	*n* = 41	[%]
Bleomycin in mg	5 (1–15)	
Number of cycles of electroporation	14 (2–62)	
Electrode types used per procedure		
Finger electrode 15 mm	21	51.2
Finger electrode 20 mm	15	36.6
Finger electrode 10 mm	4	9.8
Hexagonal electrode 30 mm	4	9.8

Therapy sessions per patient	*n* = 25	
1	16	64
2	5	20
3	2	8
4	1	4
5	1	4

### 3.3 Clinical response

A complete response was observed in 8/25 (32.0%) patients with a strong and mild reduction in 14/25 (56.0%) and 3/25 (12.0%), respectively. No patient presented with a progression of symptoms following BEST during follow-up and no non-responders were recorded. A comparison between the overall response of patients who received prior treatment, and the therapy-naïve patients showed no significant difference (W = 76.5, *p* = 0.80).

### 3.4 Imaging response

**Figure 1:**
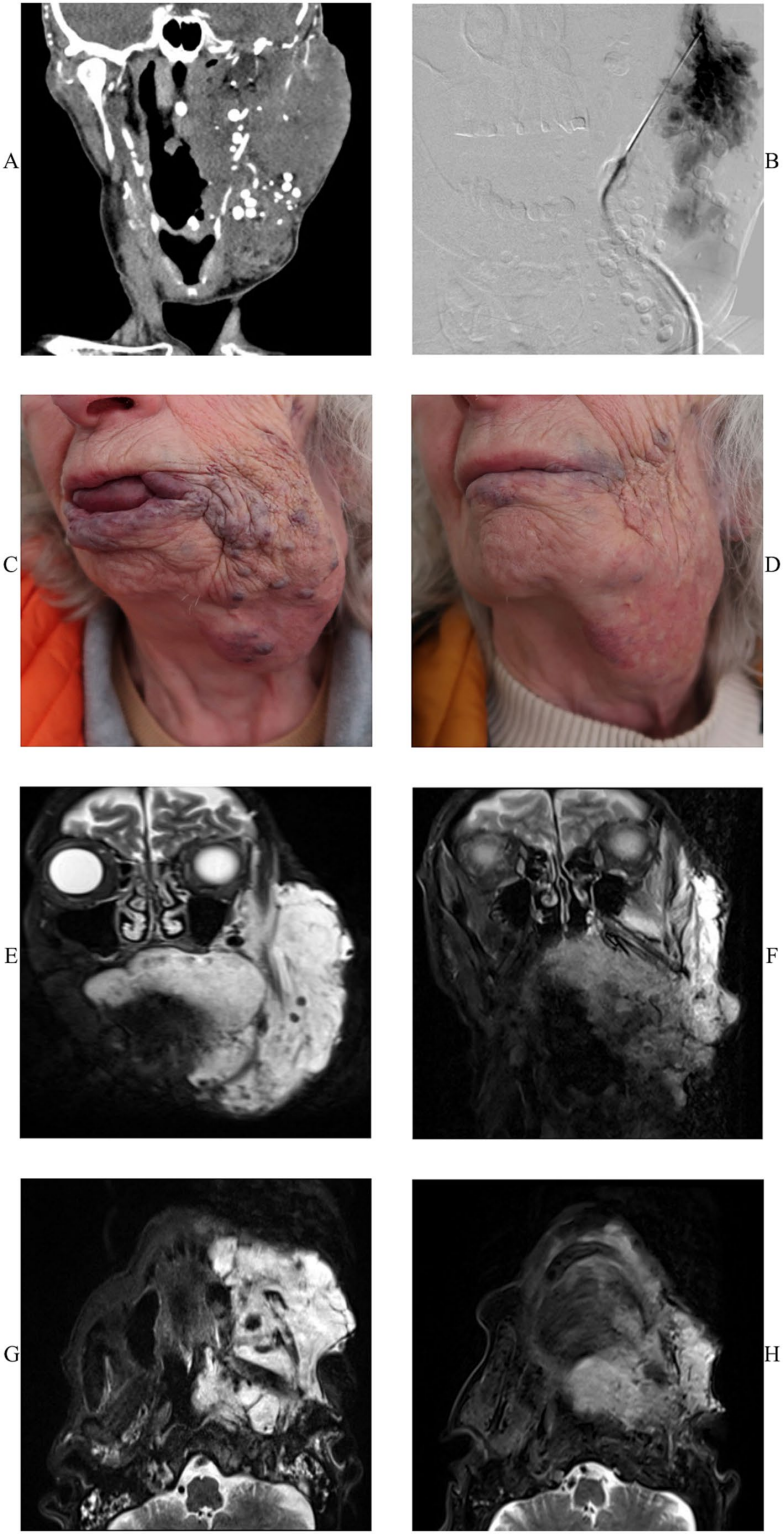
A 78-year-old patient with an extensive venous malformation (VM, indicated by arrows) undergoing two sessions of Bleomycin-Electrosclerotherapy (BEST). **(A)** Computer-tomographic angiography (CTA) of the patient demonstrating a large mass involving the left cheek with numerous phleboliths. **(B)** Digital subtraction angiography (DSA) demonstrating the distribution of contrast in one part of the venous malformation (VM). **(C)** Image of the patient before therapy demonstrating the large VM with involvement of the lips and the tongue. **(D)** Image of the same patient 8 months after the second session showing a good response with a marked response and without the typical skin discolorations following BEST. **(E,G)** T2-weighted (T2w) coronal and axial magnetic resonance (MR) images of the same patient before therapy demonstrating the extensive VM. **(F,H)** T2w coronal and axial images demonstrating a strong reduction of the treated VM.

Both pre- and postprocedural MRI was available only for 10/25 (40%) patients. Regarding the remaining 15/25 (60%) cases, 10/15 (66.7%) were paediatric patients, in whom only a clinical and US-based follow-up was performed to avoid the anaesthesia required for MRI. Figure 1 shows an exemplary case. The mean maximum volume was 451 ± 469 cm^3^ before therapy with a significant reduction to 236 ± 244 cm^3^ (*n* = 10, *W* = 45, *p* = 0.004) after BEST. Overall, 10/10 patients for whom pre- and post-treatment imaging was available presented with a lesion size reduction and 1/10 (10%) showed no residual contrast-uptake while 6/10 (60%) demonstrated a strong and 1/10 (10%) a mild reduction, respectively. No difference in contrast uptake was observed in 2/10 (20%) of cases.

### 3.5 Safety and complications

Following four sessions (4/41, 9.8%), patients reported prolonged swelling and pain for up to 6 weeks including the need for NSAR intake but without prolonged hospitalization (CIRSE grade 2). Overall, there were no major complications following the treatment of superficial SFVMs. In our patient cohort, there were no allergic reactions due to administered drugs, post-interventional syncope, or nausea/vomiting. Additionally, there were no pulmonary complications immediately or during follow-up. As an expected side effect, skin discoloration was observed after BEST in 9/25 (36%) of patients while all patients already reported partially fading during follow-up.

### 3.6 Correlation of clinical and imaging response

Using Spearman’s test, size reduction correlated moderately with clinical response even though this fails to reach significance ρ = − 0.56, *p* = 0.12.

### 3.7 Comparison of adults and children

No significant difference between clinical responses following BEST in children and adults were observed (W = 81, *p* = 0.59).

## 4 Discussion

This study, focusing on the treatment of superficial SFVM of the head and neck, demonstrates high clinical and objective success rates even in patients presenting with large as well as therapy-refractory malformations while the overall rate of complications was low and all complications were self-limiting.

A complete or at least partial response was observed in all patients which may be better than the results reported in literature for percutaneous sclerotherapy (7, 23–27). In addition, the number of treatment sessions to reach this favorable clinical outcome was low with a mean of 1.6 procedures per patient compared to 3.76 (28) and 2.1 (25) which resulted in a lower median cumulative bleomycin dosage of 7 mg in the current study compared to 32.11 mg (28) or 12 to 48 mg pingyangmycin over at least three sessions (29). No significant differences in the clinical response between children and adults could be observed in this study; however this might be due to the small cohort size. Further studies with larger cohorts will be needed to investigate this important issue.

MRI demonstrated a significant size reduction in all patients and a reduced contrast uptake in most patients. The exact relationship between clinical and imaging response is not fully clear yet: some studies reported a good correlation (30) while others do not observe a clear interaction (25, 31). The latter work suggested that in some vascular malformations, partial fibrosis or reduction of certain critical areas of the SFVM might be enough to lead to a marked reduction in symptoms; this is in line with the clinical observation that good subjective response does not always require complete imaging response. In fact, subjective complete response seems to be a more suitable therapeutic aim than complete imaging response; in fact the latter aim might even be considered counter-productive leading to unnecessary aggressive treatment of a clinical entity that is known for its tendency to recur. Consequently, MRI at follow-up should never be considered the primary or sole measure of treatment. Further research on the long-term outcome of BEST and potential differences of the recurrence rate compared to PST is needed.

The most feared complication following intravenous administration of bleomycin is pneumonitis with the potential risk for irreversible pulmonary fibrosis. So far, only anecdotal reports of clinically relevant lung injury following sclerotherapy of VMs with bleomycin or pingyangmycin have been reported (32, 33). The low rates of pulmonary toxicity may be explained on the one hand by the significantly lower overall bleomycin doses compared to the ones used in chemotherapy. Additionally, a slower pharmacokinetic uptake is being discussed. However, if intralesional compared to systemic intravenous injection really reduces the concentration of the systemic first bleomycin pass, and moreover if this is able to reduce the risk for pulmonary toxicity is yet unknown (34, 35). For now, it remains important to keep the dose of bleomycin administered as low as reasonably possible, especially given the young patient cohort.

The overall complication rate of 9.8% is similar to reports in literature on PST (7, 25, 31) and to BEST in other regions of the body (17, 36). We observed no permanent sequalae following therapy. Patients with involvement of the aerodigestive tract form a dedicated subgroup with specific risks for certain complications (18). In comparison, treatment of superficial SFVMs seems to be relatively low-risk.

The well-known side effect of postprocedural skin discoloration occurred in a relevant portion of the patients in this head and neck cohort and partially faded over the course of the observation period. This is similar to the BEST cohort by Schmidt et al. (17). While generally benign, patients need to be informed of this side-effect in advance, especially in exposed locations such as face and neck. Due to the short follow-up period, it was not possible to determine how often discolorations persist without resolution. This has to be addressed in future work focusing on long-term effects.

Another limitation of this retrospective study is the absence of standardized disease-related questionnaires to evaluate the specific symptomatology, functional impairments and disease-related quality of life in a more objective and comparable fashion.

The biggest limitation considering the evaluation of imaging response is the low rate of patients with an available post-procedural MRI due to a significant number of children reported.

The follow-up period was too short to evaluate the long-term responses which is highly relevant to allow a direct comparison of BEST and percutaneous sclerotherapy. Future studies employing an ideally randomized controlled design will be necessary to address specific questions, including whether prior treatment with conventional therapies affects BEST effectiveness and whether certain patients’ subgroups tend to respond less favorably to BEST. Currently, there are not published data on cost-effectiveness of BEST. While a single BEST session certainly produces higher costs compared to conventional sclerotherapy, the lesser amounts of sessions needed might outweigh the higher costs per session especially in larger scale malformations.

Additionally, there are currently no clear predictors of treatment response, relapse, or complications in vascular malformations, neither for conventional treatment modalities nor for novel treatment alternatives such as BEST. Appropriate stratification of patients who benefit most from individual treatment modalities is highly desirable and must be developed in a larger, multicentre setting (37).

## 5 Summary

BEST is safe and effective for treating slow-flow vascular malformations of the head and neck region. BEST has the potential to reduce the number of sclerotherapy cycles, thereby also allowing lower doses of bleomycin to achieve good to excellent subjective and objective therapy response, even in patients who were refractory to previous therapies.

## Data Availability

The raw data supporting the conclusions of this article will be made available by the authors, without undue reservation.
